# Giving patients a starring role in their own care: a bibliometric analysis of the on‐going literature debate

**DOI:** 10.1111/hex.12299

**Published:** 2014-11-04

**Authors:** Julia Menichetti, Chiara Libreri, Edoardo Lozza, Guendalina Graffigna

**Affiliations:** ^1^ Faculty of PsychologyUniversitá Cattolica del Sacro CuoreMilanoItaly

**Keywords:** bibliometric analysis, health care, patient activation, patient adherence, patient compliance, patient empowerment, patient engagement, patient involvement, patient participation, patient‐centred

## Abstract

**Background:**

Patient‐centred care has been advocated as a key component of high‐quality patient care, yet its meanings and related actions have been difficult to ascertain.

**Objective:**

To map the use of different terms related to the process of giving patients a starring role in their own care and clarify the possible boundaries between terms that are often mixed.

**Methods:**

A literature search was conducted using different electronic databases. All records containing the search terms ‘patient engagement’, ‘patient activation’, ‘patient empowerment’, ‘patient involvement’, ‘patient adherence’, ‘patient compliance’ and ‘patient participation’ were collected. Identified literature was then analysed using the Statistical Package for Social Science (SPSS). The number of yearly publications, most productive countries, cross‐concepts articles and various scientific fields dealing with the multidisciplinary concepts were identified.

**Results:**

Overall, 58 987 papers were analysed. Correspondence analysis revealed three temporal trends. The first period (2002–2004) focused on compliance and adherence, the second period (2006–2009) focused on the relationship between participation and involvement, and the third one (2010–2013) emphasized empowerment. Patient activation and patient engagement followed the temporal development trend connected to the ‘immediate future’.

**Discussion and conclusions:**

The bibliometric trend suggests that the role of patient in the health‐care system is changing. In the last years, the patient was viewed as a passive receptor of medical prescription. To date, the need to consider patients as active partners of health‐care planning and delivery is growing. In particular, the term patient engagement appears promising, not only for its increasing growth of interest in the scholarly debate, but also because it offers a broader and better systemic conceptualization of the patients’ role in the fruition of health care. To build a shared vocabulary of terms and concepts related to the active role of patients in the health‐care process may be envisaged as the first operative step towards a concrete innovation of health‐care organizations and systems.

## Introduction

The rates of chronic diseases are growing rapidly all over the world,^1^ making these kind of illnesses not only the main cause of death in the world,^2^ but also the causes of disability and decreased quality of life in most western countries.^3^, ^4^ This also implies an increased economic burden on Western Healthcare Organizations, particularly in the present period of reduced resources.^5^ Accordingly, it becomes mandatory to continuously improve health‐care system to address the long‐term, on‐going nature of chronic disease and its management. Consequently, health‐care systems all over the world claim that they are trying to reduce the cost while maintaining a higher and continuous quality of care. In 2002, the World Health Organization^6^ requested the need of every country to adopt the most appropriate and cost‐effective measures to promote healthy life, by adapting policies aimed to reduce health risks to patients’ needs. The measures, as suggested in the document, must involve patients in shared health responsibility by offering accurate information, supporting their health‐care decision, and encouraging health promotion.^6^ In line with this vision, academics and professionals agree on the importance of revising care models to make patients protagonists of their own care management.^7^ Patients need to acquire an active role in the health‐care process and its services,^8^, ^9^, ^10^ which need to become tailored to their needs and expectations.^11^ The care planning should become more patient‐centred, and patients have to be involved in the planning and delivery of health‐care services.^8^ The shared goal to innovate health‐care services and delivery should increase patients’ confidence in decision making and their awareness of health, illnesses, treatment options, symptoms and behaviours.^12^ Practically, this means improving a synergic exchange within the ‘demand’ and ‘supply’ of health‐care services.^13^ Thus, patient‐centred care has been advocated as a key component of high‐quality patient care, and the role of patient has been considered as one of the dimensions that care systems should consider to improve their patient‐centredness.^7^, ^8^ Consequently, giving patients a starring role in their health care is a widespread effort^6^, ^14^ that continues to grow, as evidenced by the continuous increase of literature on this topic.^15^ However, various terms and definitions used to capture this complex process of empowering patients to play an active role in health care (i.e., patient engagement, patient activation, patient involvement, patient participation, patient adherence, patient empowerment and patient compliance) lack agreement and shared guidelines for practice.^16^ It is clear that all these processes offer a promising pathway towards better‐quality health care, more‐efficient care and improved population health.^17^ Nevertheless, despite the growing popularity of the terms and the increasing attention by researchers, there is little consensus about what these concepts really mean and how they are related. Indeed, these different terms often have overlapping definitions. Furthermore, they are often used as synonymous or interchangeable terms to describe the pivotal role of patients in their own care. On the one hand, this tendency suggests a clear commitment shared by academics, health‐care professionals and policymakers to innovate health‐care models by giving (back) an active role to patients. On the other hand, it reveals a state of under‐determination, lack of consensus and potential confusion that may fail to drive the innovation of health‐care practice. *The objective of making patients protagonists of their care might thus be either a fashionable claim or a real goal for practice*. To offer some preliminary findings to answer this question, this study reviews the results of bibliometric analyses aimed at mapping the use of different terms related to the process of making patients protagonists of their care (i.e. patient engagement, patient activation, patient involvement, patient participation, patient adherence, patient empowerment and patient compliance). We identified studies that have been published within the last 12 years. In particular, the bibliometric analysis performed aimed to describe:

1. The trend of scientific use of each terms (number of scientific publications indexed with each of the considered terms; differences in the use of these terms in the last 12 years)
2. Country specificities (What are differences in the use of these terms across countries? What are some cultural specificities or geographical diversities in the use of these terms?)
3. Disciplinary specificities (What are differences in the use of these terms across different disciplinary fields?)
4. Interconnections and overlaps in the use of these terms (Are these terms used synonymously? What kind of associations are retrievable based on the usage of different terms?)

## Methods

We propose a bibliometric study to achieve our aims. Bibliometric analyses can provide insight into the developmental trends and the status quo of a concept or a discipline.^18^, ^19^

### Searching process

The search terms were deliberately chosen to find the widest possible range of relevant literature; specifically, we used ‘patient engagement’, ‘patient activation’, ‘patient adherence’, ‘patient empowerment’, ‘patient involvement’, ‘patient participation’ and ‘patient compliance’ as the key terms for the purpose of this research. We chose to add the word ‘patient’ to all these terms to contain the research to the health field and consider people who had to manage problematic health conditions. Our work is based on a systematic search of 4 key electronic databases (all had almost one million references; www.kcl.ac.uk/library) in the health field: Scopus, PubMed, CINAHL and ISI Web of Science.

The inclusion criteria were as follows:

1. Presence of the search key words in title/abstract/keywords (not only in the full text)
2. Only English‐written articles
3. Published in the last 12 years (2002–2013). 2002 was considered the starting year, since the World Health Organization (WHO, 2002) published a call for actions focused on the importance of active involvement of citizens as actors in their own healthy behaviours.

A standard data extraction form was developed. The data extraction form focused on the following information:

- name of the data base;
- name of the journal;
- published year;
- indexing keywords (e.g.: patient engagement);
- location/country of the first ten authors (articles with departments or institutes belonging to several geographical areas were considered as ‘multicountry’);
- departments and affiliation institution of the first ten authors (articles with departments or institutes belonging to several disciplinary fields were considered as ‘multidisciplinary’);

Data analysis was conducted using the software package for statistical analysis SPSS, version 21.0 (Armonk, NY, IBM Corp.). In addition to the descriptive statistics and cross‐tabulations of the above‐mentioned variables, we conducted a correspondence analysis (HOMALS) to summarize the variability of the observed phenomena.

### Findings

**Figure 1:**
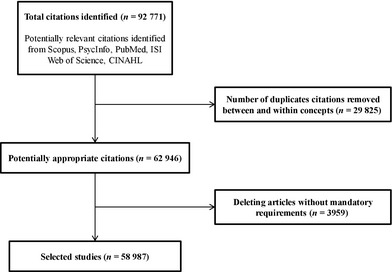
Flow diagram of literature search and selection of papers.

We retrieved 92 771 articles. After removing duplicates and excluding articles with no country or subject area information (no mandatory requirements), 58 987 articles were finally analysed (see Fig. 1).

Table 1 shows the total number of analysed articles per each concept. It is evident that patient compliance is the most widely used concept (80% of the total amount of the found), followed by patient participation (18%).

**Table 1:** Number of article per concept

Patient activation	Patient adherence	Patient compliance	Patient empowerment	Patient engagement	Patient involvement	Patient participation
280	1979	47 042	434	329	935	10 629
1%	3%	80%	1%	1%	2%	18%

We were interested in going beyond this evidence and trying to describe the interconnections between the concepts, their temporal trend and their relationships with other variables.

#### Interconnectedness of terms across publications

Our first aim was to understand whether the terms, and which terms, were used together to understand whether they were considered synonymous or whether authors referred to a specific term. Practically, we counted the number of articles referring to more than one term. As shown in Table 2, different terms did not seem to be highly related. Only two strong relationships were retrievable, as seen in the Table. Specifically, among papers indexed under the term adherence, over half of them were also indexed under the term compliance (57%). Second, among papers using the term involvement, 40% were also indexed under the term participation. However, relationships were unidirectional (only 2% of papers that focused on compliance dealt with adherence, and only 4% of papers that focused on participation dealt with involvement). Furthermore, three other interesting findings emerged. Among papers using the terms engagement, activation and empowerment, less than one fifth were also classified under participation (18, 18, and 17%, respectively). Similar percentages can suggest a conceptual relationship between the two terms.

**Table 2:** Row percentage of the interconnectedness of concepts

	Patient activation (*n* = 280)	Patient adherence (*n* = 1979)	Patient compliance (*n* = 47 042)	Patient empowerment (*n* = 434)	Patient engagement (*n* = 329)	Patient involvement (*n* = 935)	Patient participation (*n* = 10 629)	No interconnections	
Patient activation (*n* = 280)	–	1%	6%	1%	6%	2%	18%	75%	100%
Patient compliance (*n* = 47 042)	0%	2%	–	0%	0%	0%	2%	96%	100%
Patient empowerment (*n* = 434)	1%	0%	7%	–	1%	2%	17%	77%	100%
Patient engagement (*n* = 329)	5%	2%	10%	2%	–	2%	19%	70%	100%
Patient involvement (*n* = 935)	1%	0%	8%	1%	1%	–	42%	54%	100%
Patient participation (*n* = 10 629)	1%	0%	8%	1%	1%	4%	–	88%	100%
Patient adherence (*n* = 1979)	0%	–	56%	0%	0%	0%	2%	43%	100%

#### Temporal, geographical and disciplinary features of terms usage

In addition, we analysed specificities in the use of key terms by year, country, and discipline.

#### Year

First, we analysed the temporal trend of each term in relation to the percentage of indexed publications per year (Table 3). As seen before, the most frequently used terms are patient compliance (80%) and patient participation (18%). However, both terms have been used less frequently in the last few years. Between 2002 and 2013, the use of the term patient compliance has dropped by 10%. Although the use of the term patient participation has not declined (+24%) in the same years, after 2006, when the rate of its use was the highest (22%), its use started to drop slowly. The use of all other terms has shown an increasing trend, suggesting an increased interest of scholars in patient‐centred themes and a greater diversification in the terms use. Among these, the term ‘patient engagement’ has shown the greatest increase (18‐fold), followed by the threefold increase in patient activation. Moreover, if we consider 2013 (last complete year), the term patient engagement showed the highest increase in the number of indexed publications (+60%), followed by the term patient involvement (+22%).

**Table 3:** Number of publications per year (row %)

	Patient activation	Patient adherence	Patient compliance	Patient empowerment	Patient engagement	Patient involvement	Patient participation	
2002	0.2	2.4	83.2	0.6	0.1	1.3	12.3	100 (*n* = 3216)
2003	0.2	2.2	84.1	0.6	0.1	1.0	11.7	100 (*n* = 3781)
2004	0.3	2.6	82.8	0.5	0.1	1.7	12.0	100 (*n* = 3918)
2005	0.3	2.5	78.1	0.4	0.2	1.3	17.2	100 (*n* = 4627)
2006	0.3	2.9	71.9	0.8	0.2	1.7	22.2	100 (*n* = 4669)
2007	0.4	2.8	76.1	0.7	0.3	1.3	18.5	100 (*n* = 5656)
2008	0.2	3.5	73.4	0.5	0.4	1.4	20.5	100 (*n* = 5519)
2009	0.7	4.1	72.3	0.7	0.2	1.3	20.8	100 (*n* = 5025)
2010	0.6	3.3	75.7	0.8	0.6	1.6	17.3	100 (*n* = 5909)
2011	0.5	4.0	75.3	0.6	0.6	1.7	17.2	100 (*n* = 6514)
2012	0.7	3.3	74.9	0.9	1.0	1.5	17.6	100 (*n* = 6762)
2013	0.8	3.9	75.2	1.0	1.8	2.1	15.3	100 (*n* = 6032)
% 2013–2002	+388	+65	−10	+66	+1867	+62	+24	

#### Country

North America has the highest percentage of literature indexed with the considered terms (42%), followed by Europe (28%) and the ‘multicountry’ literature (16%) (Table 4). As shown in Table 4, patient activation (64%), patient engagement (58%) and patient adherence (50%) are largely present in North American literature, whereas patient involvement (51%) and patient empowerment (47%) are mostly used in literature published in Europe. The other terms, however, did not show significant differences from the average trend (total number of articles across countries).

**Table 4:** Terms usage across countries

	North America	Europe	Asia	Africa	Oceania^a^	South America	Multicountry	
Total	24 770 (42%)	16 309 (28%)	4855 (8%)	687 (1%)	2382 (4%)	650 (1%)	9334 (16%)	
Patient activation	64	17	7	–	2	–	10	100
Patient adherence	50	22	6	2	3	1	16	100
Patient compliance	42	27	9	1	4	1	16	100
Patient empowerment	32	47	6	0	3	1	11	100
Patient engagement	58	20	1	0	7	–	13	100
Patient involvement	23	51	4	0	4	0	17	100
Patient participation	44	31	4	1	6	1	14	100

a The term is here used to denote the continent comprising Australia and proximate islands (Melanesia, Polynesia, Micronesia).

#### Subject area

Table 5 reports the use of considered key terms across disciplines, showing that most studies that used these terms were conducted in the field of medicine (*n* = 39 370). However, medicine had most publications in general. Different terms showed a homogeneous use across all the disciplines, without notable differences (Table 5).

**Table 5:** Disciplines percentage within concepts

	Medicine	Nursing	Psychology	Other health sciences	Physical and life sciences	Pharmacology and toxicology	Social sciences	Multidisciplinary	
Total	39 370 (67%)	3989 (7%)	1457 (3%)	1756 (3%)	5506 (9%)	4548 (8%)	1916 (3%)	445 (1%)	
Patient engagement	69	8	2	5	8	6	2	0	100
Patient activation	70	6	2	4	10	4	4	1	100
Patient adherence	68	6	2	3	9	8	3	1	100
Patient empowerment	65	7	2	2	10	9	4	1	100
Patient involvement	69	5	2	3	10	9	2	0	100
Patient participation	66	7	2	4	9	8	3	1	100
Patient compliance	67	7	3	3	9	8	3	1	100

#### Correspondence analysis

**Figure 2:**
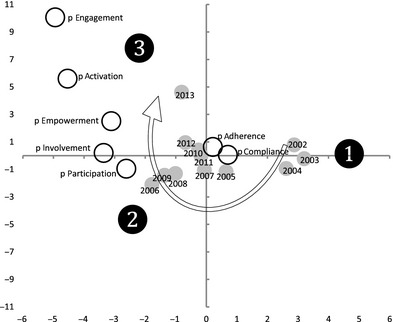
Two‐dimensional solution of multiple correspondence analysis. The map shows the positions and association between the variables.

Since the analysis of countries and disciplinary areas revealed similar distribution for all terms, we chose to perform correspondence analysis using – as active variables – the year of publication and the key terms used in the publications. The analysis yielded two dimensions (explained inertia *x*‐axis = 0.53 and *y*‐axis = 0.51) that allowed us to define and compare temporal development of the terms and to map them. Figure 2 clearly shows a four‐step temporal development.

The first period of the temporal trend (2002–2004) seems to be more connected to patient adherence and patient compliance (these two terms are also interconnected, as we saw in the previous paragraph on ‘Interconnectedness of terms among publications’). The most frequently used terms in the second period (2006–2009) were patient participation and patient involvement. In the third period (2010–2013), patient empowerment was the most frequently used term. Finally, two terms, patient activation and patient engagement, were not specific to any particular year; instead, their use followed the temporal development of the literature debate (i.e. the ‘immediate future’), thus showing a possible improved usage in the future.

## Discussion

This study presented a bibliometric analysis of journal articles published between 2002 and 2013 in the health field, which debated the role of patients in their health‐care management. The bibliometric analysis aimed to describe the use of different terms (i.e. patient engagement, patient activation, patient involvement, patient participation, patient adherence, patient empowerment and patient compliance) concerning the active role of patients in their health‐care management to unveil actual trends and tendencies in the on‐going literature debate.

Overall, the term compliance was the most widely used term in the identified literature, as it is probably one of the oldest concept developed in the field.^20^ The analysis showed that different terms analysed in this study have been only partially interconnected during the last twelve years. Although all terms refer to the intention of making patients actors of their care, they were rarely used together in literature (with the exception of patient adherence and patient compliance, patient involvement and patient participation). These data suggest that each term has a specific meaning related to a clear action. Our bibliometric analysis unveiled a specific time trend in the use of these terms, indicating a clear development in the emphasis on the role of patients in their care management, which can be clarified through different axis (individual vs. relational, process vs. outcome, active vs. passive).

Historically, patients have to follow the suggestions of health professionals and be ‘executors’ of their care, with a limited freedom and participation in the decision making. Compliance and adherence were indeed mentioned mainly in the literature published in the first period (2002–2004), which focused on the importance of recognizing the role of patients in health management from a patient‐centred medicine perspective. As our interconnections analysis confirmed, adherence and compliance are related concepts in the literature, and both focus mainly on the behavioural components of patients’ experience. Furthermore, they appear to share a classic vision of patient as a passive ‘executor’ of medical prescriptions.^21^, ^22^ Thus, those terms consider mainly an individual context of care. Then, in the second period (2006–2009), the focus seemed to be on the role of relational and contextual elements of the clinical encounter (i.e. communication strategies) in sustaining the active role of patients in their health management. Most typical for this period were the terms ‘participation’ and ‘involvement’, which theorise the importance of the relational characteristics of the clinical consultation in improving shared decision making.^23^, ^24^ In this sense, the attention is on the dyadic context of care. In the third period [2010–2012], the term ‘empowerment’ became the most popular, emphasizing the shared sensibility about the importance of considering the complexity of subjective patients’ experiences and of sustaining patients’ autonomy and self‐determination in care management. Empowering patients gives patients a subjective sense of control over their disease, thus gives them an enhanced responsibility in care management. Finally, the emergent tendency related to the most recent years [2013–…] suggests further development towards considering patients as critical stakeholders in the planning, delivery and evaluation of health‐care services. Specifically, the near future of the health‐care debate seems to focus on the concept activation and even more on engagement (which is the term with the highest rate of growth). These concepts are linked to a consumer behavioural perspective that considers patients as subjects involved in a specific cultural and social context. This may suggest an increased attention to the different dimensions (not only subjective, but also contextual, relational and organizational) that may foster or hinder patients’ ability to truly become protagonist of their care.

Thus, the analysis showed that different terms, although they all imply the need to make patients actors of their care, convey very different representation of the patients and of their roles. This ‘panacea’ of terms, the conceptual margins of which are sometimes overlapping in the literature debate, could disorient the experts interested in innovating the health‐care system by making patients more protagonists of their care journey^25^ and hinder real practice. A shared and grounded conceptualization of the different possible roles that patients may assume in their care management may improve interventions created to innovate the health care.

**Figure 3:**
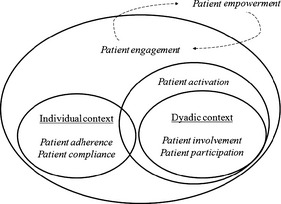
A framework to understand relationships among terms.

In particular, the term patient engagement appears promising, not only for its increasing growth of interest in the scholarly debate, but also because it offers a broader and better systemic conceptualization of the patients’ role in the fruition of health care. Thus, it can be defined an ‘umbrella term that qualifies the systemic relationship that occurs between the ‘supply’ and the ‘offer’ of health care – at different levels and in different situation’^26^. The concept of patient engagement temporally and conceptually overtakes other terms, which are more traditionally used to generally denote the role of patients in their care (see Fig. 3). The concept of activation has some conceptual overlap with engagement, although the two concepts differ according to the breadth of the patient–health‐care relationship considered. The concept of activation is mainly limited to the prototypical situation of a doctor–patient consultation, while the concept of engagement seeks to consider multiple levels of the patients’ relationship with the health‐care system. Moreover, adherence and compliance appear more narrow in their conceptualization of the patient role and of his/her exchange with the health care compared to the concept of engagement. First, these concepts (i.e. adherence, compliance) show a hierarchical vision of the health‐care relationship, where the health‐care provider (i.e. the expert) prescribes to the patient (i.e. the lay actor) the rules to better manage his/her disease. These concepts imply an evaluation of the patients’ attitudes and behaviours, as more or less close to a gold standard.^27^, ^28^ Otherwise, the concept of engagement shows a democratizing vision of the exchange between demand and supply of health services. Furthermore, it also takes into account the subjective, emotional and motivational aspects of such exchange. The concepts of involvement and participation (often used as synonymous) refers primarily to the dyadic rapport of the medical consultation and to the cognitive and emotional component of shared clinical decision making. The link between these concepts and the concept of engagement is evident because all these concepts theorize that the patients should be able to negotiate the stipulation of their care. Nevertheless, these concepts are mostly limited to the dyadic context of the exchange between doctor and patient, whereas the concept of engagement is related to a broader and systemic exchange between demand and supply of health services, where clinical consultation is only one of the possible levels of the analysis. In this framework, the concept of empowerment appears more intertwined with the concept of engagement. The concept of empowerment connotes the activity of patients made possible through the fostering of a sense of control over their disease.^29^ In this sense, empowerment is a pre‐requisite of patient engagement, which is fostered by achievement and good experiences of patients on their health‐care journey.

## Limitations of the study

Some limitations of this study must be considered. Overall, a bibliometric analysis represents only one view of scientific debate on this topic. Thus, our results were extracted and inferred from structural data of papers that included the selected terms. Further studies need to be conducted to deepen the literature contents and boundaries of these terms. Furthermore, the analysis is based on English‐articles, although papers on this topic may be published in other languages as well. Finally, it should be considered that making patients protagonists of their care is a final goal of a process in which every stakeholder (patient, doctor, carers…) has specific roles and responsibilities. The problem of making patients protagonists in their health management is very challenging, different care should be given to different patients and different stages of their treatment. Moreover, particular situations (e.g., cultural barriers, unhealthy lifestyles, drug addictions) may raise doubts about whether the aim of engaging patients should be pursued and how. Further studies need to investigate how to manage those situations.

However, the present study offers a wide and accurate overview of the emergent themes related to patients’ role in health care, providing useful insights into how to better conceptualize this theme; thus, it may orient the future debate on this issue.

## **Conclusions**

The definitions and the historical trend of all these terms (i.e. patient engagement, patient activation, patient involvement, patient participation, patient adherence, patient empowerment and patient compliance) clearly show the presence of specific characteristics and differences between apparently similar concepts. The indiscriminate use of all these terms reflects a lack of clarity of what health‐care systems need to do to achieve the important goal of making patients protagonists of their care. The bibliometric trend shows that the role of patient into the health‐care system is changing. In the last years, the patient was conceived as a passive treatment participant, there is a growing need today to consider patients as active partners of health‐care planning and delivery. However, there is still a lack of a clear conceptualization able to translate this shift into clinical practice. We propose to consider the specific semantic role of these terms, because every term has a practical consequence in health‐care practice. To build a shared vocabulary of terms and concepts related to the active role of patients in the health‐care process may thus be envisaged as the first operative step towards a concrete innovation of health‐care organizations and systems.

In this framework, the term patient engagement appears particularly promising, not only for its increasing growth of interest in the scholarly debate, but also because it offers a broader and better systemic conceptualization of the patients’ role in health care. From this perspective patient, engagement may offer theoretical as well as pragmatic insights to innovate organizational strategies aimed at improving the effectiveness and efficiency of health care.^30^ We suggest that these strategies should be able to face the current societal challenges, include a clear perspective on the patients’ role into clinical practice, and consider different levels of sustainability and applicability (subjective, organizational, and economic)

## Conflicts of interest

No conflicts of interest have been declared

## Source of funding

No source of funding have been declared
